# Affordable and real-time antimicrobial resistance prediction from multimodal electronic health records

**DOI:** 10.1038/s41598-024-66812-5

**Published:** 2024-07-16

**Authors:** Shahad Hardan, Mai A. Shaaban, Jehad Abdalla, Mohammad Yaqub

**Affiliations:** 1 Mohamed bin Zayed University of Artificial Intelligence, Abu Dhabi, UAE; 2 Sheikh Khalifa Medical City, Abu Dhabi, UAE

**Keywords:** Machine learning, Antimicrobial resistance

## Abstract

The spread of antimicrobial resistance (AMR) leads to challenging complications and losses of human lives plus medical resources, with a high expectancy of deterioration in the future if the problem is not controlled. From a machine learning perspective, data-driven models could aid clinicians and microbiologists by anticipating the resistance beforehand. Our study serves as the first attempt to harness deep learning (DL) techniques and the multimodal data available in electronic health records (EHR) for predicting AMR. In this work, we utilize and preprocess the MIMIC-IV database extensively to produce separate structured input sources for time-invariant and time-series data customized to the AMR task. Then, a multimodality fusion approach merges the two modalities with clinical notes to determine resistance based on an antibiotic or a pathogen. To efficiently predict AMR, our approach builds the foundation for deploying multimodal DL techniques in clinical practice, leveraging the existing patient data.

The discovery of penicillin was the starting point of antibiotic usage that led to the treatment of many diseases and reduced the number of deaths from infections^1^. However, the increase in the prescription of broad-spectrum antibiotics and the inadequate usage of antibiotics are diminishing their effectiveness and increasing the resistance of microbes toward them. As a result, antimicrobial resistance (AMR) causes clinical and economic drawbacks, emphasizing the importance of its early identification. AMR is expected to be the greatest threat in healthcare by 2050, forecasting around 10 million deaths if not controlled. In addition, it is currently considered a hidden pandemic^2^. For high-income countries, it is anticipated that the number of annual deaths from AMR becomes 2.4 million between 2015 and 2050^3^. Worldwide actions taken so far prove that there is room for successful prevention and control of resistance patterns.

Currently, when an antibiotic prescription decision is to be made, the process of acquiring the susceptibility results and the accompanying steps in the healthcare system are timely^4^. In some areas of the world, antibiotics are consumed without any cultures taken beforehand. Moreover, other faster methods such as the assessment from DNA are expensive, not widely accessible, and require additional data collection. However, artificial intelligence (AI) advancements within the healthcare framework pave the way for several faster, affordable, and handy technologies to improve human lives. Machine learning (ML) solutions are applied to various problems including diagnosis of infections and early identification of diseases. Tackling the AMR problem from an ML perspective focuses on four different areas: predicting AMR on a patient’s level, managing antibiotic prescription, aiding clinical decision support systems, and considering environmental factors^5^. This work involves the prediction of AMR in an affordable and real-time approach, as data-driven medication prescription and infection prevention may provide better control of the prevalence of AMR.

From the previous studies on AMR using ML,^6^ uses ICU EHR data to predict AMR, employing statistical analysis and machine learning models, nevertheless, their ML methodologies necessitate the manual selection of clinical features, resulting in an insufficient understanding of bacterial transmission within the ICUs. Another study^7^ constructs a decision algorithm to recommend the most suitable antibiotic for urinary tract infection (UTI) patients based on resistance predictions, successfully reducing the use of second-line antibiotics. Another investigation^8^ focuses on antibiotic exposure prediction using different tiers of data, demonstrating that larger and more complex datasets yield higher accuracy. However, the reliability of the study’s findings heavily relies on the quality of the data. Finally, an AutoML approach^9^ is employed to predict antibiotic resistance in hospitals using oversampling techniques to handle the class imbalance, raising concerns about noise generation in the medical domain. A more recent study^10^ uses time-series deep-learning methods to predict AMR for Methicillin-resistant Staphylococcus aureus-positive cultures, proving the efficiency of their approach on two different datasets.

Unlike the previous work on AMR, our data-driven approach aims to (1) seamlessly integrate with current clinical platforms while utilizing existing data, and (2) provide predictive outcomes instantly to help support and speed up clinical decision-making. This makes our proposed method (1) affordable as it does not require additional tests or interventions, (2) accessible as it could be easily embedded within a hospital workflow, and (3) real-time as it produces instant predictions on a regular and continual basis. Our proposed method uses deep learning techniques on the three modalities available in the electronic health records (EHR) data. We utilize the existing multimodality fusion mechanisms to provide a robust ground for the exploration and efficiency of predicting AMR using the most suitable configurations and features extracted from the data. To the best of our knowledge and based on an extensive literature review, our research represents the first attempt to address the problem of AMR using deep learning affordable, accessible, and real-time methods on multimodal EHR data aiming to benefit the microbiology field from the AI advancements.

The increase in the adoption of EHR platforms in hospitals led to acquiring more patient data. These datasets include an abundance of information including patients’ admissions, diagnoses, hospital procedures, and clinical notes. The type of data collected is multimodal and can be divided into three modalities: (1) time-invariant such as age and gender, (2) time-series such as lab results, and (3) clinical notes taken by healthcare providers. ML models can use either type or fuse these types for classification tasks. Most studies that use EHR data did not focus on all three modalities available. However, authors in^11^ leveraged the three modalities on two tasks: diagnosis prediction and acute respiratory failure. They proved that fusing them outperforms state-of-the-art models that do not utilize them collectively. Their results showed the importance of including clinical notes along with structured data in EHR models, as well as the significance of the fusion model. Taking their study forward, we explored the potential of using deep learning multimodal techniques for predicting AMR.

The complexity of the AMR problem requires great efforts from both the clinical and the ML aspects to support the field in combating the problem. There are various effects that AMR could have on a patient’s health which differs according to the patient’s medication history as well as the surrounding environment and populations. Thus, the need for personalized early identification of AMR supports clinical practitioners in prescribing effective medications. Since only a few works have been done to understand this problem from an ML perspective using EHR data, we aim to explore suitable approaches toward building machine learning models that efficiently predict AMR from a patient’s history. The study potentially improves the understanding of ML for AMR and contributes to more effective strategies for combating this global health threat. The main contributions of this work can be summarized as follows:

- We formalize an affordable, real-time, and accessible method that tackles the prediction of AMR using deep learning multimodality techniques.
- We assess the prediction performance based on multiple criteria such as the amount of data, the imbalance in datasets, and time-series features. This assessment is based on the decision-making processes related to patients in the ICU stay.
- We leverage the readily available and inexpensive EHR data to make predictions using the patients’ history to potentially aid clinicians in decision-making by providing prompt insights regarding AMR.

## Overall framework

**Figure 1:**
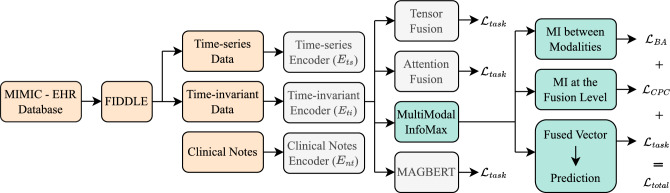
Description of the pipeline followed in this work. We start with the raw EHR data, which is then reformulated using the FIDDLE framework to two modalities: time-invariant and time-series, along with clinical notes. The three modalities are encoded and passed to four different fusion mechanisms. Among them is MMIM, which involves three losses for three different tasks and achieves superior results compared to other fusion methods. MMIM maximizes the mutual information at the input modalities level, as well as at the fusion level. A fusion network is used to predict the resistance.

Our approach follows the framework described in Fig. 1, which is partly inspired by^11^. It first takes the raw EHR data, which is then preprocessed using the FIDDLE pipeline^12^. The extracted records include information about the ICU stays and the medical history of the patients collected at the related hospital admission before transfer to the ICU, and they come in three modalities: time-series, time-invariant, and clinical notes. We follow two approaches when performing the prediction classification task: resistance of pathogens (infecting patients) tested against a certain antibiotic or patients infected with a certain pathogen. Then, the modalities in the dataset are encoded depending on their type. A linear layer encodes the time-invariant data, while ClinicalBert^13^ encodes the clinical notes. For the time-series data, the model uses LSTM, StarTransformer^14^, or the transformer encoder. After encoding, four modality fusion mechanisms are investigated. We first explored the one that outperformed in^11^ which is the multimodal adaptation gate (MAGBERT)^15^ using BERT^16^. We also explored the tensor fusion^17,18^ and attention fusion^19^ mechanisms. The MultiModal Infomax (MMIM)^20^ mechanism, showing a greater potential on our data, maximizes the mutual information between inter- and intra-modality fusion data. To achieve that, it uses three losses to optimize each of the following tasks: predicting AMR using $\mathcal {L}_{task}$, understanding the inter-modality mutual information (MI) using $\mathcal {L}_{BA}$, and the intra-modality MI using $\mathcal {L}_{CPC}$. Our code is available at https://github.com/shahdhardn/abr-ehr.

## Results

### Cohort

The dataset used is MIMIC-IV^21^ which is an EHR database that includes data for patients at the Beth Israel Deaconess Medical Center in the critical care units from 2008-2019. The tables used in this study have a variety of data types. From the available time-invariant data, we use information about demographics, admissions, ICU stays, diagnosis, and procedures. These tables also include data regarding the medical history of the patient before admission to the ICU. From the tables including time-series data, we use microbiology events, input and output events, lab events (non-microbiology), charted events, and procedure events. The clinical notes include a concatenation of the discharge and radiology notes in MIMIC-IV. They are tokenized using the WordPiece approach in^22^. Our framework involves extracting a cohort that acquires susceptibility tests during their ICU stays. The cohort is then used to predict one of two outcomes: resistance or susceptibility. We apply the FIDDLE^12^ data preprocessing pipeline. FIDDLE^12^ enables flexible extraction and definition for the cohort studied by transforming and filtering the MIMIC data. First, the tables are modified to create an input that fits the FIDDLE framework. Then, FIDDLE includes three main steps: pre-filtering to remove rare variables, transforming the input into time-series and time-invariant matrices, and post-filtering these matrices. The uniqueness of FIDDLE lies in reducing the number of decisions the user should make, making fewer assumptions about data, and dealing with missing values in a way that suits the nature of EHR data through the carry-forward method. The original work is applied to the MIMIC-III database and is modified in this work to fit MIMIC-IV. The rest of this section describes findings from data exploration and structuring. More insights from FIDDLE are described in Sect. 5.1.

**Figure 2:**
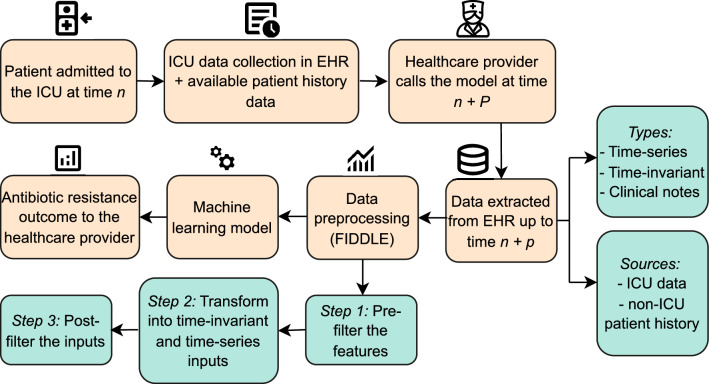
This Figure shows the healthcare setting in which our approach can be used to support antibiotic stewardship programs in hospitals.

Given the approach taken to define the cohort, Fig. 2 shows our general approach in a clinical setting for any patient admitted at time *n*. The prediction from our machine learning model can be extracted at time $n + P$, where *P* is in (*n*, *D*), and *D* is the discharge time. We note that, later, we will use *T* as the time when the outcome is predicted as this is the time that the lab order is derived from the MIMIC dataset. In a clinical setting, the healthcare provider can trigger the prediction at any time after the admission as long as there is enough patient data to use for the model.

### Data exploration

**Figure 3:**
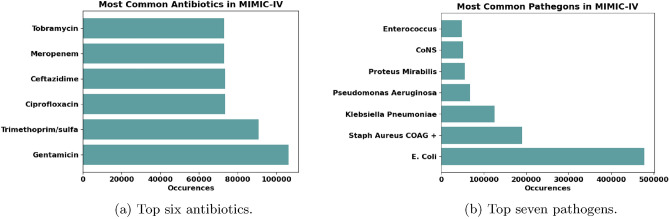
The most common antibiotics and pathogens present in the MIMIC-IV dataset.

Regarding the microbiology data, MIMIC-IV has 27 types of antibiotics and 647 types of pathogens. It also includes the type of culture samples acquired from patients, with urine cultures having the highest rate. To investigate the most suitable approach for predicting AMR, our study considers assigning resistance labels from two perspectives: patients infected with the same pathogen and patients tested against the same antibiotic. Studying patients infected with the same pathogen aims to understand if the strength of the pathogen’s resistance can give us useful conclusions about the patient’s reaction. The exploration starts by observing the availability of these antibiotics or pathogens in the dataset. Figure 3a presents the top six antibiotics prescribed, with Gentamicin being the most frequently prescribed, and is therefore considered for further analysis. Additionally, Fig. 3b shows the top seven pathogens according to the data availability. To examine the most suitable pathogen for the model, we studied the imbalance factors, starting from the ICU stays having the first three common pathogens in Fig. 3b. Due to the nature of the problem in which the antibiotic resistance cases are far less than the susceptibility cases, the issue of imbalance arises. Imbalance is an important factor to consider in models so that the model’s decision is not skewed towards the most represented category over the least represented one, thus making them unreliable. The analysis shows that their imbalance rate is high, which refers to the low resistance rates against the antibiotics prescribed for these pathogens. Thus, the focus of this study is on *P. aeruginosa* as it considers the trade-off between the number of ICU stays and the imbalance rate. According to this choice of antibiotic and pathogen, we create separate datasets using FIDDLE considering each factor when creating the labels.

### Datasets after merging modalities

During the study on Gentamicin, we focused more on three of the time-series settings according to the prediction time ($T=3$, $T=4$, and $T=10$). Since FIDDLE outputs matrices for each modality, we need to merge them to prepare the input for the ML models. The merging step reduces the number of ICU stays as it considers those who have data in all three modalities. For $T=3$, the number of time-series input features is 6,095, the number of time-invariant input features is 70, the number of ICU stays is 1,322, and the imbalance factor is 12.35. For $T=4$, time-series input features: 5,992, time-invariant input features: 72, ICU stays: 1,245, imbalance factor: 11.45. For $T=10$, time-series input features: 7,038, time-invariant input features: 67, ICU stays: 1,287, imbalance factor: 14.30. Furthermore, the study on *P. aeruginosa* included a time-series input of size 4924, a time-invariant input of size 69, and an imbalance factor of 3.15.

To understand the effect of the multimodality technique, we ran different types of models on only the time-invariant data, only the time-series data, only the text data, and all three modalities. We experimented on several datasets belonging to the AMR problems with different parameters for the prediction time and time granularity.

### Baseline models

For the antibiotic resistance task, we mainly experimented with the dataset that includes patients who undertook a susceptibility test for the Gentamicin antibiotic for MIMIC-IV. The first dataset considers a prediction time of 4 and a time granularity of 1. We used a batch size of 20, a learning rate of 1e−4, BERT size of 768, and binary cross-entropy (BCE) with logits and class weights as the loss function. Table 1 describes the results on each modality and the fusion model of the Gentamicin dataset ($T=4, dt=1$). Using MAGBERT as an attention mechanism, StarBert achieved the highest performance, reaching an AUROC of approximately 0.69 and an AUPR of 0.16. Also, Since data imbalance impacts the models’ effectiveness, we analyzed it more on this dataset as can be found in Supplementary Fig. 2.

The second Gentamicin dataset considers a prediction time of 3 and a time granularity of 1. We experimented with a batch size of 20, a learning rate of 5e−5, and a BERT size of 768. Table 1 represents the results on the modalities alone along with the fusion model using MAGBERT. On this dataset, BertEncoder provided the best performance, with an AUROC of 0.61 and an AUPR of approximately 0.15. With almost the same model configuration, we experimented with the Gentamicin ($T=10, dt=1$) dataset which has an imbalance ratio of 14.3. But, since the imbalance rate is the highest for this setting, results are not sufficient for further experimentation.

Furthermore, since the problem also tackles the prediction of AMR from the pathogen perspective, the best results using the same encoders are shown in Table 1. The rest of the combinations do not yield good results, so they are excluded from the analysis. As can be observed, BertStar achieves the best performance reaching an AUROC of 0.63 and an AUPR of 0.2407.

**Table 1:** Result on the MIMIC-IV datasets on the test set with the weighted BCE loss and a batch size of 20. The fusion models use the MAGBERT mechanism. The table also presents the average AUROC along with its confidence interval (CI).

Data and Settings	Models	AUPR	AUROC	$\overline{\text {AUROC}}$	CI
Gentamicin $T = 4, dt=1$ learning rate: $1e-4$	Line	0.0886	0.5027	0.5980	(0.5439, 0.6521)
	LSTM	0.1136	0.5852		
	Star	0.1274	0.5893		
	Encoder	0.1151	0.5547		
	BERT	0.1644	0.5807		
	BertLstm	0.1445	0.6077		
	BertStar	**0.1631**	**0.6874**		
	BertEncoder	0.1249	0.6224		
	LstmBert	0.1422	0.6156		
	StarBert	0.1379	0.6495		
	EncoderBert	0.1364	0.5824		
Gentamicin $T=3, dt= 1$ learning rate: $5e-5$	LSTM	0.0935	0.4659	0.5388	(0.4818, 0.5958)
	Star	0.1041	0.5173		
	BERT	0.1513	0.4927		
	BertLstm	0.0935	0.4659		
	BertStar	0.1271	0.6103		
	LstmBert	0.1395	0.5770		
	StarBert	0.1223	0.5883		
	BertEncoder	**0.1474**	**0.6145**		
	EncoderBert	0.1041	0.5173		
*P. aeruginosa* $T=3, dt=1$ learning rate: $1e-5$	LSTM	0.2166	0.5000	0.5775	(0.5500, 0.6050)
	BERT	0.2120	0.6000		
	BertLstm	0.2405	0.5800		
	BertStar	**0.2407**	**0.6300**		

### Comparison of fusion mechanisms

The same datasets are used to assess the change in the fusion models. The results are shown in Table 2 including the outcome of attention fusion, tensor fusion, and MMIM. For the Gentamicin task, the tensor fusion outperforms the attention fusion. Also, the tensor fusion outperforms MAGBERT when LSTM is the time-series encoder. Giving the best performance, MMIM achieved an AUROC of approximately 0.76 and an AUPR of 0.1825 on the Gentamicin ($T=4, dt=1$) dataset. Similarly, MMIM outperforms using the Gentamicin ($T=3, dt=1$) dataset obtaining an AUROC of 0.66 and an AUPR of 0.227. On the *P. aeruginosa* dataset, MMIM achieved the best AUROC of 0.69, while the tensor fusion mechanism attained the highest AUPR of 0.30. Regarding the confidence intervals (CI) for the results mentioned in Tables 1 and 2, we found that both the lower and upper limits of CI for AUROC are around ±0.05 for Gentamicin and ±0.03 for P. aeruginosa.

**Table 2:** Results of the three attention mechanisms implemented as alternatives to MAGBERT. The Table includes the outcomes of the Gentamicin and the *P. aeruginosa* tasks as well as the average AUROC for each cohort is shown with its confidence interval (CI). “Attn” refers to the attention fusion mechanism, “Outer” refers to the tensor fusion mechanism, “MMIM” refers to Multimodal Infomax, “W.BCE” refers to weighted binary cross entropy, “C.E.” refers to cross entropy. All the Gentamicin experiments are done with a learning rate of $5e-5$, while the *P. aeruginosa* ones used a learning rate of $1e-5$.

Data	Model	Loss	AUPRC	AUROC	$\overline{\text {AUROC}}$	CI Interval
Gentamicin $T=4, dt=1$	LstmBertAttn	W.BCE	0.1988	0.6459	0.6198	(0.5695, 0.6701)
	BertLstmAttn	W.BCE	0.0623	0.3391		
	LstmBertOuter	W.BCE	0.1602	0.6686		
	StarBertOuter	W.BCE	0.1852	0.6306		
	LstmBertMMIM	C.E.	**0.1825**	**0.7565**		
	StarBertMMIM	C.E.	0.1596	0.6782		
Gentamicin $T=3, dt=1$	LstmBertOuter	W.BCE	0.2209	0.5996	0.6225	(0.5699, 0.6752)
	LstmBertMMIM	C.E.	**0.2270**	**0.6555**		
	StarBertMMIM	C.E.	0.1869	0.6125		
*P. aeruginosa* $T=3, dt=1$	LstmBertOuter	W.BCE	**0.3075**	0.6400	0.6650	(0.6398, 0.6902)
	LstmBertMMIM	W.BCE	0.2731	**0.6900**		

## Discussion

The approach implemented in this study aims to explore the effect of fusing the different modalities of EHR on the performance of the AMR classification tasks. Using multimodal deep learning techniques captures more complex patterns in the rich EHR data that includes a variety of types and structures. Thus, different fusion techniques present in other applications can be investigated on EHR tasks to predict AMR.

### Performance analysis on the AMR task

We experimented with three different prediction times for the Gentamicin dataset and one for the *P. aeruginosa* dataset. Starting from the Gentamicin dataset, with the prediction time of 4 hours and considering time-series data alone, the time-series encoders fail to achieve a high AUROC or AUPR. Using BERT only for text data leads to approximately the same results as the time-series data. When merging the data and using the MAGBERT fusion mechanism, the AUROC increases by 10% when applying StarBert. Table 2 shows that implementing the MMIM fusion mechanism achieves the best performance on the Gentamicin AMR task. The loss used in this experiment is weighted cross entropy which helps reduce the effect of class imbalance. It is also noticeable in the AMR tasks that the trade-off between AUROC and AUPR differs between models. This behavior is normal with highly imbalanced data, where the low number of positive predictive values leads to a low AUPR but the true positive rate and the false positive rate are still good enough to lead to a high AUROC.

### Performance analysis on the Gentamicin task

With a prediction time of 3 hours and a time granularity of 1 hour, we noticed a decrease in classification performance compared to the prediction time of 4 hours. Using one modality leads to low AUROC that cannot be considered in a clinical setting. When implementing fusion mechanisms, the performance increases for some models. Using MAGBERT, BertEncoder achieved the best performance by having an AUROC of 0.6145 and an AUPR of 0.1474. BertStar also acquired a similar AUROC but with a lower AUPR. Other than LstmBert, models where the main modality is time series resulted in a lower AUROC than when the clinical notes are the main modality. The tensor fusion mechanism on the same dataset led to a higher AUROC for the LstmBert model than LstmBert with MAGBERT. Regarding MMIM, it achieved the best AUROC on the Gentamicin ($T=3, dt=1$) data, reaching an AUROC of 0.6555 and an AUPR of 0.227 with LSTM as a time-series encoder. In general, after merging the three modalities, the prediction time of 3 results in both a lower number of patients and fewer time-series features compared to a prediction time of 4. Thus, it is expected that the performance will be lower than that of the dataset Gentamicin ($T=4, dt=1$). Experimenting on the Gentamicin dataset that has a prediction time of 10 hours led to a weak performance of the models. The reason is due to the high imbalance in the dataset. We observe that, for the AMR task, patients’ data from the susceptibility tests are mostly acquired in the first few hours of their ICU stay. This shows that imbalance has a higher effect on the performance of the AMR classification task than the number of time-series or time-invariant extracted from the MIMIC database.

### Performance analysis on *P. aeruginosa*

We only examine the time-series encoders of LSTM or Star. The results show a similar pattern to Gentamicin’s dataset in which the multimodality approach, and especially MMIM, achieves higher performance than a single modality. The highest performance is an AUROC of 0.69 by LstmBert using the MMIM fusion mechanism and an AUPR of 0.30 by the tensor fusion mechanism. The AUPR in the case of the *P. aureginosa* dataset is higher than all the Gentamicin models as its imbalance rate is significantly lower. However, our limited analysis for predicting on the basis of the pathogen is because it is less informative to clinical practitioners under this framework. The reason it is less applicable is because it provides less knowledge about the type of antibiotic that should be prescribed to the patient, and is thus, a less personalized decision.

### Fusion mechanisms

In total, there are four fusion mechanisms used in this work. Among them, the attention fusion fails to learn properly the different modalities in the EHR data due to its difference in nature from the work’s original task: multimodal sentiment analysis (MSA). Since we do not have synchronized data similar to the case in MSA, the model cannot attend to different modalities effectively. Compared to the attention fusion mechanism, tensor fusion performed well and was close to MMIM. The reason is that tensor fusion works by creating different regions for each modality and for each combination of modalities as a result of the outer product. Thus, it produces a high dimensional space for the embeddings to provide a better understanding of the interaction between modalities.

Due to the hierarchical nature of the MMIM approach, it led to better AUROC values. The MMIM method takes into consideration the mutual information both present among the different modalities and between the prediction results and the fused data. Also, it is efficiently able to make use of time-invariant data. On the other hand, the other three fusion mechanisms only account for the relationship between the time series and clinical notes. The consideration of only two modalities is because these fusion mechanisms give equal attention to each modality, which is not applicable in the EHR case. The time-invariant data can be, in some cases, less valuable in making predictions than the time series or clinical notes.

## Conclusion

The importance of the AMR medical case requires a thorough analysis and exploration of EHR data and machine learning models. As the EHR data is huge and contains a lot of valuable information, we found the FIDDLE pipeline the most feasible method for the MIMIC database. FIDDLE leverages the existence of different modalities and types of data in EHR databases. Moreover, the existence of different modalities in the MIMIC data allows the adoption of multimodality fusion models. Out of the fusion mechanisms adopted in this study, MMIM worked the best for the AMR prediction task for both cases of prediction according to the antibiotic and the pathogen. Our analysis shows that the difference in the prediction time for time-series data plays a major role in the performance of the model as it determines the size and imbalance factor of the dataset. Generally, our work uses advanced machine learning techniques to set a new milestone in the field of predicting antimicrobial resistance. The intersection between artificial intelligence and microbiology is narrow, which requires extensive efforts from researchers to reach deployable clinical solutions.

Further work can expand our understanding of AMR by exploring more antibiotics or pathogens in the EHR data. To make the application more feasible in clinical practice, the solution can be integrated with a medication prescription recommendation algorithm. Additionally, the framework can be extended to the outpatient setting by considering the historical data from the patient’s visits, as well as to the non-ICU inpatient setting. Technically, the models can consider time-series data differently by using irregular time-series encoders.

## Methods

### Insights from FIDDLE

Since FIDDLE is a flexible framework, we adjust it to fit the AMR task. In this study, there is a greater focus on creating a dataset according to the antibiotic compared to the pathogen. However, similar details follow when creating the *P. aeruginosa* dataset. Generally, when preparing for the AMR task, the pipeline should extract a new ICU stays dataset. First, we use the original microbiology events MIMIC-IV table and choose patients having results for the antibiotic sensitivity test. We only take records labeled as sensitive or resistant, excluding those with pending or intermediate statuses. Then, we extract records belonging to one type of antibiotic to use them as resistance labels for the classification task. In our case, Gentamicin is considered since it has the largest amount of data. After that, the microbiology table is merged with the ICU stays table on the patient and admission IDs. For the Genatmicin cohort, this process reduces the number of ICU stays from around 76,000 to 13,658. A total of 12,442 ICU stays had a sensitive result and 1,216 had a resistant result, having an imbalanced ratio of 91/9, or approximately 10. We then add the label information to the onset hour from the microbiology events tables.

Studying the cohort having a sensitivity result for Gentamicin further, most patients have their results during the first hours of their stay. This insight is described in Supplementary Fig. 2. Regarding the length of stay, the highest frequency is for ICU stays of almost 25 to 30 hours. Around 24% of the patients stayed in the ICU for over 200 hours.

Moving to the dataset of patients infected with *P. aeruginosa*, a similar analysis is done. The result is a dataset that includes information related to 2,103 ICU stays with an imbalance rate of 3.14, which is much smaller in size than Gentamicin’s dataset. For patients who are infected with *P. aeruginosa*, their prescriptions include five types of antibiotics: Cefepime ($\approx$9%), Meropenem ($\approx$89%), Piperacillin/Tazobactam ($\approx$1.4%), Ciprofloxacin ($\approx$0.38%), and Ceftazidime ($\approx$0.05%). Similar to the Getanimicin cohort, the *P. aeruginosa* cohort also has the patients’ cultures’ results during the first hours of the ICU stay.

The next step is the inclusion criteria, in which we filter the data according to the prediction hour set for each task. We first start by removing data for children under 18 years old, followed by removing data of patients who die or are discharged before the prediction hour. After excluding death and discharge cases, the summary statistics for the Gentamicin $T=4$ dataset are shown in Supplementary Table 2. Then, patients who have a resistance result during the period [0, *T*], where T is the onset hour, are given a label of 1, and 0 otherwise. From our analysis presented in Supplementary Table 1, patients had their lab cultures taken in the first hours which assisted in the choice of T for the tasks.

As discussed, the work in^11^ works by combining three different modalities from EHR data: time-invariant, time-dependent, and clinical notes. Setting *B* as the batch size, the time-invariant data is denoted by ${\textbf {I}}_{ti} \in \mathbb {R}^{B \times D_{ti}}$, the time-series data by ${\textbf {I}}_{ts} \in \mathbb {R}^{B \times L \times D_{ts}}$, and the tokens IDs of the clinical notes by ${\textbf {I}}_{nt} \in \mathbb {R}^{B \times D_{nt}}$. Here, *L* refers to the timestamps and represents *T*/*dt*, $D_{ti}$ to the dimensions of time-invariant features, $D_{ts}$ to the dimension of the time-series features, and $D_{nt}$ to the length of the clinical notes.

To encode the time-invariant data, a linear fully-connected layer is used, followed by a ReLU activation layer, ${\textbf {E}}_{ti} = ReLU(Linear({\textbf {I}}_{ti}))$, where ${\textbf {E}}_{ti} \in \mathbb {R}^{B \times D'_{ti}}$ and $D'_{ti}$ is the dimension of the time-invariant encoded feature. To encode the time-series data,^11^ proposes different approaches, all following the equation

1 $$\begin{aligned} {\textbf {E'}}_{ts}&= ENC({\textbf {T}}_{ts}), \nonumber \\ {\textbf {E}}_{ts}&= ReLU(Linear({\textbf {E'}}_{ts})), \end{aligned}$$

Here, ${\textbf {E'}}_{ts} \in \mathbb {R}^{B \times L'}$, where $L'$ is the hidden size, and ${\textbf {E}}_{ts} \in \mathbb {R}^{B \times D'_{ts}}$, where $D'_{ts}$ is the number of neurons in the network. The time-series encoders can be LSTM, StarTransformer, or the original transformer encoder. Finally, the weights of the ClinicalBERT model presented in^13^ are used to encode the clinical notes tokens. The encoded notes are described as ${\textbf {E}}_{nt} = ClinicalBERT({\textbf {I}}_{nt})$, where ${\textbf {E}}_{nt} \in \mathbb {R}^{B \times D'_{nt}}$. Even though other large language models were developed after ClinicalBERT and trained on medical data, ClinicalBERT remains useful in our context as it relies on the MIMIC-III dataset, which directly fits our tasks. At this stage, all modalities are ready to be fused.

### Fusion mechanisms

The four fusion mechanisms discussed in this section are originally established for multimodal sentiment analysis (MSA). In MSA, there are three modalities: text, visual, and audio. All three are synchronous and have a high impact on understanding human sentiment. Since this is not the case for EHR data, as different modalities have different impacts on the medical task, we adapt the fusion strategy accordingly.

We considered the fusion mechanism that is taken as the outperforming one in^11^ as the baseline, which is multimodal attention gating (MAGBERT)^15^ using BERT. We also adopted three other fusion mechanisms: tensor fusion^17,18^, attention fusion^19^, and Multimodal InfoMax (MMIM)^20^. Attention fusion considers the long-range dependencies between the modalities by having one transformer per modality and computing the cross-attention between them. Tensor fusion investigates the aspects of unimodal, bimodal, and trimodal representations by calculating the outer product between modalities. We will provide more details about the approach of fitting MMIM to the EHR data since it obtains the best results. Further explanation regarding the other three mechanisms can be found in their original works or in^11^.

#### Fusion with hierarchical mutual information maximization

MMIM works by maximizing the mutual information between inter- and intra-modality data^20^. Since the approach adopts the mutual information (MI) technique, the issue of the intractable MI bounds has to be tackled for higher computational efficiency. The authors in^20^ utilized a variety of parametric and non-parametric methods to estimate the true values of the MI bounds. The architecture of the model takes the unimodal representations of the three modalities and passes them into two parts. The first part is the fusion network that leads to the prediction result, while the second implements MI for the input and fusion levels. After that, the losses produced from the prediction and MI tasks are back-propagated to increase the learning of the model.

**Maximizing MI at the input level:** By assuming a correlation between the two modalities: X and Y, the mutual information is then estimated as follows

2 $$\begin{aligned} {\begin{matrix} I (X,Y) &{} = \mathbb {E}_{p(x,y)} \left[ \log \frac{q(y|x)}{p(y)} \right] + \mathbb {E}_{p(y)} \left[ KL(p(y|x) || q(y|x)) \right] \\ &{} \le \mathbb {E}_{p(x,y)} \left[ \log q(y|x)\right] + H(Y) \\ &{} \triangleq I_{BA}, \end{matrix}} \end{aligned}$$

where *H*(*Y*) is the differential entropy of Y. In^20^, it is shown that the text modality dominates and gives higher performance when considered as the main modality as it has a higher dimension. Thus, we consider the clinical notes as X and pair it with Y which is either the time-series or time-invariant data. In this case, we optimize two MI bounds for the two pairs of modalities. To approximate *q*(*x*|*y*), we follow^23^ that considers it as a multivariate Gaussian distribution $q_\theta ({\textbf {y}}|{\textbf {x}}) = \mathcal {N} ({\textbf {y}}| \varvec{\mu }_{\theta _1}({\textbf {x}}), \varvec{\sigma }^2_{\theta _2}({\textbf {x}}) {\textbf {I}})$, where $\theta _1$ and $\theta _2$ are the parameters for the two neural networks that predict the mean and the variance, respectively. For optimizing the approximation of *q*(*y*|*x*) for the two pairs of modalities using likelihood maximization, we utilize the same loss function available in^20^. To compute the entropy term *H*(*Y*), we use the Gaussian Mixture Model (GMM) by constructing two normal distributions for the two classes. In case of the antibacterial resistance task, we have $\mathcal {N}_S(\varvec{\mu }_1, \varvec{\Sigma }_1)$ and $\mathcal {N}_R(\varvec{\mu }_2, \varvec{\Sigma }_2)$, where $\varvec{\mu }$ is the mean, $\varvec{\Sigma }$ is the covariance matrix, and *S* and *R* denote the sensitive and resistance classes. Having a sufficiently large sample, the entropy of a multivariate normal distribution is given by

3 $$\begin{aligned} H = \frac{1}{2} \log \left( (2\pi e)^k det(\varvec{\Sigma }) \right) , \end{aligned}$$

where the GMM vectors dimensionality is denoted by *k* and the determinant of the covariance matrix $\varvec{\Sigma }$ is denoted by $det(\varvec{\Sigma })$. When applying this approach to the MSA task, we consider that the two classes, positive and negative, have almost equal weights (prior probabilities), leading to a certain computation of the entropy adopted in^20^. However, since the EHR tasks are highly imbalanced, we cannot assume equal weights for both distributions. Also, for some training iterations, it will not be possible to calculate $det(\varvec{\Sigma }_2)$ since the number of occurrences of the underrepresented class in one batch could lead to having a negative determinant. Therefore, assuming imbalanced weight and that the distributions of the two classes are disjoint, we take the lower bound as an approximation and calculate the entropy as

4 $$\begin{aligned} H(Y) = \omega _S \log (det(\varvec{\Sigma }_1)), \end{aligned}$$

where $\omega _S$ is the weight (prior probability) of the sensitive class. More details of the derivation are present in^20,24^. Finally, the optimization of the MI lower bound maximization at the input level is given by the following loss function

5 $$\begin{aligned} \mathcal {L}_{BA} = - I_{BA}^{nt, ts} - I_{BA}^{nt, ti}, \end{aligned}$$

**Maximizing MI at the fusion level:** The main goal of fusing the three modalities is to perceive modality-invariant information. This is done by maximizing the MI between the modalities and their fused outcome. Following^25^ and^20^, we adopt Contrastive Predictive Coding (CPC) as a way to predict the representations from the outcome of the fusion. We apply the Euclidean norm on the prediction produced by a neural network $F_\phi$ with parameters $\phi$ as well as the true vector $h_m$ where $m \in \{ti, ts, nt\}$. The neural network is applied on the vector $Z = g(E_{ti}, E_{ts}, E_{nt})$, where *g* is the fusion network. After that, we estimate their correlation as follows

6 $$\begin{aligned} s(h_m, Z) = \exp \left( {\frac{h_m}{||h_m||_2} \left( \frac{F_\phi (Z)}{||F_\phi (Z)||_2}\right) ^T}\right) , \end{aligned}$$

This score is then used to compute the Noise-Contrastive Estimation loss between the fusion outcome and each modality. Each representation is considered as a positive point and all other representations in the same batch are considered as negative. Denoting the representation in one batch as ${\textbf {H}}_m$, we calculate the NCE loss according to

7 $$\begin{aligned} \mathcal {L}_N(Z, {\textbf {H}}_m) = - \mathbb {E}_{\textbf {H}} \left[ \log \frac{s(Z, h_m^i)}{\sum _{h_m^j\in {\textbf {H}}_m} s(Z, h_m^j)} \right] , \end{aligned}$$

Therefore, the CPC loss for the fusion level is calculated as

8 $$\begin{aligned} \mathcal {L}_{CPC} = \mathcal {L}_N(z, ti) + \mathcal {L}_N(z, ts) + \mathcal {L}_N(z, nt), \end{aligned}$$

Combining all three losses, the total loss during training is computed as

9 $$\begin{aligned} \mathcal {L}_{total} = \mathcal {L}_{task} + \alpha \mathcal {L}_{CPC} + \beta \mathcal {L}_{BA}, \end{aligned}$$

where $\alpha$ and $\beta$ dictate the effect provided to the MI maximization (both kept as 0.1), and $\mathcal {L}_{task}$ is the cross entropy loss. Figure 1 explains the overall approach.

### Supplementary Information

Supplementary Information.

## Data Availability

The MIMIC-III data utilized in this work is publicly available through PhysioNet (http://mimic.physionet.org).
