# Supplementary material for: Affordable and real-time antimicrobial resistance prediction from multimodal electronic health records
Source: Sci Rep. 2024 Jul 16;14:16464. doi: 10.1038/s41598-024-66812-5 (PMC11252127; doi:10.1038/s41598-024-66812-5)
Supplement: Supplementary file 1 — Supplementary Information. [file 41598_2024_66812_MOESM1_ESM.pdf]

Affordable and Real-time Antimicrobial Resistance Prediction from Multimodal Electronic Health Records - Supplementary Information

1 More on the Datasets

Table S1: The application of the inclusion criteria for different tasks and prediction time specifically for the Gentamicin cohort. The Table describes how the higher the prediction time, the lower the number of ICU stays extracted. Note that the original number of ICU stays is 13,658 for antibiotic resistance against Gentamicin. We also investigated the data extracted for the *P. aeruginosa* pathogen, giving a similar pattern.

**Prediction time (hr)**	**Size after excluding death**	**Size after excluding discharges**	**Final size**
1.0	13,653	13,633	5,884
2.0	13,651	13,617	4,588
3.0	13,646	13,586	3,816
4.0	13,642	13,560	3,311
10.0	13,588	13,383	2,137
12.0	13,576	13,300	1,950

Table S2: The summary statistics of the Gentamicin task dataset.

Population	Gentamicin,$T = 4$
Size	3,311
Median age	66
Gender: F(%)	49.5
Race: Black	375
Race: White	2,133
Race: Hispanic	113
Race: Asian	79
In hospital death(%)	15.5
Median LOS	102

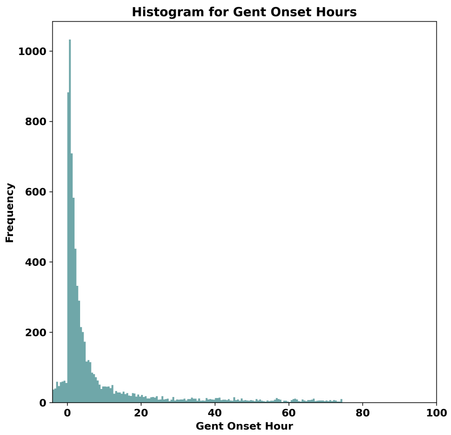

Fig. S1: The distribution of the number of ICU stays with respect to the Gentamicin sensitivity test onset hour.

2 Handling class imbalance

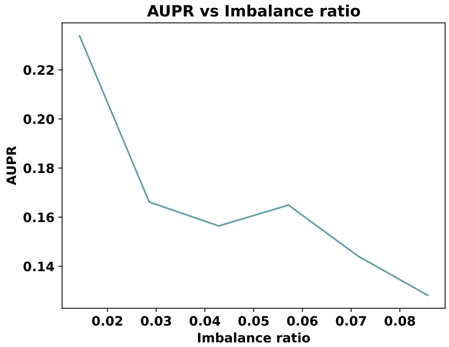

Fig. S2: To obtain superior results with respect to the AUPR score, the dataset should have a lower imbalance ratio since AUPR takes into account the precision-recall trade-off, which is highly sensitive to class imbalance [1]. Thus, the undersampling technique [2] is investigated in this Figure to help reduce the imbalance ratio by randomly selecting a subset of samples from the majority class. The Figure indicates that the AUPR score declines when the imbalance ratio expands. The technique was conducted on the Gentamicin (T = 4, dt = 1) using the BertLstm model.

References

[1] Saito, T., Rehmsmeier, M.: The precision-recall plot is more informative than the roc plot when evaluating binary classifiers on imbalanced datasets. PloS one **10**(3), 0118432 (2015)

[2] Liu, X.-Y., Wu, J., Zhou, Z.-H.: Exploratory undersampling for class-imbalance learning. IEEE Transactions on Systems, Man, and Cybernetics, Part B (Cybernetics) **39**(2), 539–550 (2008)
